# Dehiscences and fenestrations: methodological care necessary to avoid errors in diagnosis and measurement

**DOI:** 10.1590/2177-6709.22.5.025-029.oin

**Published:** 2017

**Authors:** Alberto Consolaro

**Affiliations:** 1 Full Professor, School of Dentistry, Bauru, São Paulo State. Full Professor, Graduate School of Dentistry, Ribeirão Preto - University of São Paulo.

**Keywords:** Dehiscences, Fenestrations, Gingival recessions, Orthodontic movement

## Abstract

The low prevalence of gingival recessions observed in orthodontic clinical practice may be assigned to the fact that in studies in which dehiscences and bone fenestrations are described as frequent, they were diagnosed based on: 1) dry skull studies; 2) areas with periosteal reflection together with flap; and 3) imaging techniques with low sensitivity to detect these defects, which have a delicate structure and function. In areas of pseudo-dehiscences and fenestrations, the periosteum and the alveolar cortical bone are very thin; also, they either have been removed during preparation of the dry specimens in the areas for analysis, or, alternatively, have not been investigated using an ideal imaging method.

## RESUMO

A ausência de prevalência elevada das recessões gengivais relacionadas à prática clínica ortodôntica se explica, provavelmente, porque, nos trabalhos em que as deiscências e fenestrações ósseas são descritas como frequentes, essas foram diagnosticadas a partir de: 1) estudos em crânios secos; 2) áreas com rebatimento periosteal junto com o retalho; e 3) uso de métodos imagiológicos que falham em sensibilidade para captá-las, pela sua delicadeza estrutural e funcional. Nessas áreas de pseudodeiscências e fenestrações, existe periósteo e cortical óssea alveolar muito fina, que foram eliminados nos procedimentos de preparação dos espécimes secos, nas áreas para a análise ou, então, não se aplicou um método imagiológico ideal.

Since the first uses of CT scanners for orthodontic and orthopedic diagnosis and treatment planning, a question has been raised in several investigations: *Does tooth movement, particularly toward the buccal surface, promote dehiscence and fenestration in the thin cortical bone plate and, thus, increase the chances of gingival recession during treatment or after its completion?*

The first published studies that attempted to answer this question used medical CT scanners and found no significant lack of alveolar cortical bone in the teeth that had been moved and in the expanded palates. However, these outcomes should always be carefully analyzed because, in fact, the absence of buccal bone, indicating the presence of dehiscences and fenestrations, may result from the difficulty in detecting them when using medical CT imaging methods.

Other studies, which used cone beam CT, more sensitive and adequate for oromaxillofacial tissues, have attempted to analyze the same association, suggesting the occurrence of dehiscences and fenestrations. Laudable attempts have been made to create software and applications to increase the sensitivity of imaging methods in the evaluation of cortical bone, particular in the buccal bone plate.

There seems to be a contradiction here: if dehiscences are frequent, why are gingival recessions not as frequent in orthodontic clinical routine? Why are gingival recessions not representative of common and prevalent consequences in patients that undergo orthodontic treatment? 

Several steps should be taken before we conclude that dehiscences and fenestrations increase after orthodontic movement or orthopedic procedures in the maxilla. These steps are associated with previous knowledge of biology and anatomy of the periodontal region and maxillary bones in the buccal and lingual surfaces, as well as of the application of this knowledge to the day-to-day practice in Dentistry.

Therefore, this study explores knowledge, findings and procedures that may lead to insights for further basic and clinical investigations under these observation prisms. 

## PREVIOUS KNOWLEDGE

**Figure 1:**
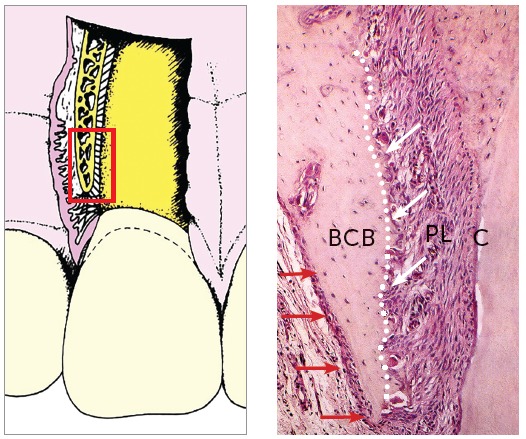
Buccal bone formed by very thin buccal cortical bone (BCB) and bundle bone (white arrows) not always detected in imaging studies. Red arrows show periosteum. PL = periodontal ligament: C = cementum (HE, 25 X magnification, murine tooth).

**Figure 2:**
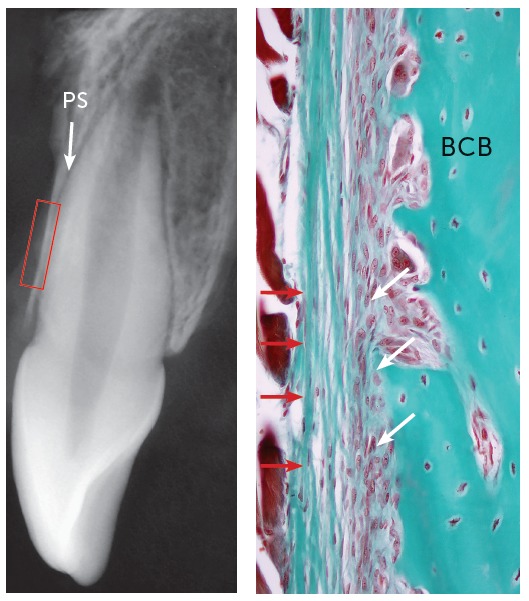
Periosteum formed by external fibrous layer (red arrows) and internal cellular layer (white arrows) directly associated with buccal cortical bone plate (BCB). PS = periodontal space (TM, 40x, human tooth).

1.1) Buccal cortical bone is usually extremely thin (Figs 1 and 2); sometimes it is thinner than the periodontal ligament, which ranges from 0.2 to 0.4 mm, which corresponds to two or three strands of hair; that is, it is extremely thin. Under these conditions, its low level of bone mineralization and insufficient thickness preclude, in most cases, the generation of images using the CT scanning or radiography resources that are clinically available today.
1.2) Constant normal bone remodeling^1^ precludes cumulative mineralization, necessary to generate a detectable image in the more delicate alveolar cortical bone. In addition to being very thin, most mineralized structures in the buccal cortical bone have low levels of mineralization, which makes it difficult to obtain detectable images.
1.3) There are two sources of nutrients and cells in the free cortical bone: the periodontal ligament and the periosteum.^1^ ^,^ ^2^ ^,^ ^3^ The periosteum has two layers (Figs 1 and 2):
  » an dense and fibrous external layer, of collagen and a few fibers, through which soft tissue, muscle and other structures insert into bone;
  » an internal, highly cellular and vascularized layer crossed by collagen fibers that fuse with bone collagen to form the Sharpey fibers, whose function is to ensure insertion, continuity and fixation of the periosteum to the external bone surfaces. This fixation is so firm that specific surgical instruments are required to detach the periosteum from the cortical surface.
1.4) In some cases, the clinical impression is that some teeth do not have an alveolar buccal cortical plate. However, this plate is, in fact, very thin and covered by the periosteum, a true envelope or external bone lining. On the buccal surface, teeth and this very thin cortical and little mineralized cortical plate are associated with the periodontal ligament in the usual way. This explains the large number of cases in which we have the impression that there is no cortical bone or periosteum, but that, despite that, do not develop gingival recessions.
1.5) Gingival recessions occur when the periosteum reacts to the lack of well-formed buccal bone, which does not develop because of the inflammatory agents that may be acting in the region.^3^ Among these agents, we find bacterial plaque, incorrect brushing and the most common causes of occlusal trauma: occlusal abnormalities, premature contacts and bruxism.
1.6) When there is no inflammatory agent or inflammation, the periosteum on the buccal or lingual surfaces forms larger bone layers and preserves its function, which responds to the demands of covering and protection, as well as distribution of forces in the alveolar cortical plate. Inflammation induces an acid environment and accumulation of mediators that induce bone resorption. The environment necessary for bone neoformation and remodeling should be neutral or basic, that is, should be free of inflammation due to bacteria or trauma.
1.7) Every time the periosteum, which usually is attached to the internal surface of flaps, is reflected during surgeries, osteocyte nutrition is removed from the more superficial layers of the cortical bone and necrotize inside their minute individual cavities.

When these cavities do not have cells during bone remodeling, the bone in the region is resorbed and, immediately after that, a new matrix develops and reconstitutes the part that was temporarily lost. The thickness of this resorption, due to periosteum reflection, ranges from 0.1 mm to 1-2 mm, depending on the region and on other anatomic and surgical variables.

Because of this possible consequence, and to avoid post-surgical dehiscences and fenestrations, the periodontist should, when observing that the alveolar cortical bone is thin and delicate, choose the split flap technique, in which the periosteum remains firmly attached to the surface of the buccal bone.

## IMPORTANT PREVIOUS PROCEDURES

2.1) Dry crania and mandibles should not be used in any study about dehiscences and fenestrations. At the time crania are prepared, soft tissues are removed, which results in the removal of the periosteum that was firmly imbricated and attached to cortical bone. 

During preparation, the removal of the periosteum results in the concurrent removal of the thin cortical bone attached to it, which produces defects, similar to dehiscences and fenestrations, with irregular and serrated margins and that, in fact, are false or non-existent. True natural dehiscences and fenestrations have a regular, not serrated or brittle contour; their margins are rounded and clearly limited. 

A very important basic factor in the study of bones is that margins and edges are always rounded and never angled. In all procedures, either surgical or not, that result in angles, bone tends to become rounded in a few weeks. 

2.2) Similarly and for the same reasons, occurrence and measurements should not be evaluated during surgery because, when the surgeon reflects the periosteum, the thinner cervical layers of alveolar cortical bone are moved together with the periosteum that is attached to the flap. Buccal cortical bone may be compared to a thin eggshell after boiling.

In these cases, dehiscences are seen as also irregular, with serrated, broken or segmented edges, measuring more than what they should actually measure had the flap and the periosteum not been reflected. Moreover, pseudo-dehiscences and fenestrations may be falsely detected as the inevitable result of raising and reflecting the periosteum during surgery.

## HOW TO INVESTIGATE WITHOUT THESE METHOD LIMITATIONS 

As a suggestion for the studies about dehiscence and fenestration frequency and measurement, the most adequate method includes more sensitive observation procedures or microscopic analyses of areas where the periosteum has not been surgically reflected or removed for the preparation of dry crania for anatomic studies. 

The detection of these images requires very fine sensitivity of CT scanners and other forms of image acquisition. Buccal periosteum is normal in comparison to other areas, but alveolar cortical bone on the buccal and lingual surfaces are very thin and have little mineralized material, which, moreover, is under constant remodeling.

The use of transverse anatomic cuts of human cadaver heads to evaluate the mucosa, the periosteum and the alveolar cortical bone *en bloc*, together with the buccal surfaces of teeth, may be a solution for such limitation. A still more accurate method is analysis of these cadaver specimens using CT microscanners, whose sensitivity is greater than that of cone beam CT scanners.

## FINAL CONSIDERATIONS

In sum, the low prevalence of gingival recessions found in orthodontic clinical practice may be explained by the fact that, in studies that describe bone dehiscence and fenestration as frequent, they have been diagnosed based on:

1) dry skull studies;
2) areas where the periosteum was reflected together with the flap; and
3) imaging studies whose sensitivity was low to detect these defects, which have a delicate structure and function.

In fact, these areas of pseudo-dehiscences and fenestrations had a periosteum and a very thin alveolar cortical bone plate, which were removed during specimen preparation for analysis, or the imaging method used was not ideal. To ensure that results reflect clinical facts, we should select samples that are adequately prepared and methods that are more sensitive and accurate.
